# Supplementary material for: Learning to detect AI texts and learning the limits
Source: PLoS One. 2025 Oct 15;20(10):e0333007. doi: 10.1371/journal.pone.0333007 (PMC12527182; doi:10.1371/journal.pone.0333007)
Supplement: S2 File — All data and scripts used in the study, some additional descriptive statistics, scalable formate figures. (ZIP) [file pone.0333007.s002.tgz › data and scripts/OSF/scripts/analysis/mixed models jamovi/outputs/questionaire_correctness_all.pdf]

Generalized Linear Model

Model Info

Info	Value	Comment
Model Type	Linear	Classical Regression/ANOVA
Call	glm	Correctness ~ 1 + `Demographics: ai_improve` + `Demographics: ai_risk` + `Demographics: ai_quality` + `Demographics: ai_lacks_creativity` + `Demographics: ai_lacks_understanding` + `Demographics: ai_grammar` + `Demographics: ai_avoidance` + `Demographics: ai_dumbing` + `Demographics: ai_fascination`
Link function	Identity	Coefficients in the same scale of y
Distribution	Gaussian	Normal distribution of residual
R-squared	0.0636	Proportion of reduction of error
AIC	1304.0900	Less is better
BIC	1343.0010	Less is better
Deviance	2314.7507	Less is better
Residual DF	244	
Chi-squared/DF	9.4867	Overdispersion indicator
Converged	yes	Whether the estimation found a solution

Model Results

Loglikelihood ratio tests

	X^2	df	p
Demographics: ai_improve	3.69709	1	0.055
Demographics: ai_risk	7.93e-4	1	0.978
Demographics: ai_quality	0.60676	1	0.436
Demographics: ai_lacks_creativity	0.01773	1	0.894
Demographics: ai_lacks_understanding	0.01032	1	0.919
Demographics: ai_grammar	6.84723	1	0.009
Demographics: ai_avoidance	0.17708	1	0.674
Demographics: ai_dumbing	0.01796	1	0.893
Demographics: ai_fascination	0.00247	1	0.960

Parameter Estimates

Names	Estimate	SE	95% Confidence Interval		exp(B)	95% Exp(B) Confidence Interval		z	p
			Lower	Upper		Lower	Upper		
(Intercept)	10.20866	0.193	9.82988	10.587	27137.218	18580.728	39634.00	52.8237	< .001
Demographics: ai_improve	0.30906	0.161	-0.00598	0.624	1.362	0.994	1.87	1.9228	0.056
Demographics: ai_risk	-0.00375	0.133	-0.26487	0.257	0.996	0.767	1.29	-0.0282	0.978
Demographics: ai_quality	0.11801	0.152	-0.17893	0.415	1.125	0.836	1.51	0.7789	0.437
Demographics: ai_lacks_creativity	0.01889	0.142	-0.25909	0.297	1.019	0.772	1.35	0.1332	0.894
Demographics: ai_lacks_understanding	0.01370	0.135	-0.25051	0.278	1.014	0.778	1.32	0.1016	0.919
Demographics: ai_grammar	0.37730	0.144	0.09470	0.660	1.458	1.099	1.93	2.6167	0.009
Demographics: ai_avoidance	0.05052	0.120	-0.18477	0.286	1.052	0.831	1.33	0.4208	0.674
Demographics: ai_dumbing	-0.01658	0.124	-0.25911	0.226	0.984	0.772	1.25	-0.1340	0.893
Demographics: ai_fascination	-0.00864	0.174	-0.34936	0.332	0.991	0.705	1.39	-0.0497	0.960
